# Hardware-Efficient Phase Demodulation for Digital *ϕ*-OTDR Receivers with Baseband and Analytic Signal Processing

**DOI:** 10.3390/s25103218

**Published:** 2025-05-20

**Authors:** Shangming Du, Tianwei Chen, Can Guo, Yuxing Duan, Song Wu, Lei Liang

**Affiliations:** 1 Sanya Science and Education Innovation Park of Wuhan University of Technology, Sanya 572000, China; shangming21@whut.edu.cn (S.D.); guocan@whut.edu.cn (C.G.); 307489@whut.edu.cn (Y.D.); 307396@whut.edu.cn (S.W.); 2 School of Safety Science and Emergency Management, Wuhan University of Technology, Wuhan 430070, China; chentianwei@whut.edu.cn; 3 National Engineering Research Center of Fiber Optic Sensing Technology and Networks, Wuhan University of Technology, Wuhan 430070, China; 4 School of Computer Science and Artificial Intelligence, Wuhan University of Technology, Wuhan 430070, China; 5 School of Mechanical and Electronic Engineering, Wuhan University of Technology, Wuhan 430070, China

**Keywords:** distributed acoustic sensing, FPGA, RFSoC, *ϕ*-OTDR, heterodyne detection, phase demodulation, Hilbert transform, wavelet, interferometric fading

## Abstract

This paper presents hardware-efficient phase demodulation schemes for FPGA-based digital phase-sensitive optical time-domain reflectometry (ϕ-OTDR) receivers. We first derive a signal model for the heterodyne ϕ-OTDR frontend, then propose and analyze three demodulation methods: (1) a baseband reconstruction approach via zero-IF downconversion, (2) an analytic signal generation technique using the Hilbert transform (HT), and (3) a wavelet transform (WT)-based alternative for analytic signal extraction. Algorithm-hardware co-design implementations are detailed for both RFSoC and conventional FPGA platforms, with resource utilization comparisons. Additionally, we introduce an incremental DC-rejected phase unwrapper (IDRPU) algorithm to jointly address phase unwrapping and DC drift removal, minimizing computational overhead while avoiding numerical overflow. Experiments on simulated and real-world ϕ-OTDR data show that the HT method matches the performance of zero-IF demodulation with simpler hardware and lower resource usage, while the WT method offers enhanced robustness against fading noise (3.35–22.47 dB SNR improvement in fading conditions), albeit with slightly ambiguous event boundaries and higher hardware utilization. These findings provide actionable insights for demodulator design in distributed acoustic sensing (DAS) applications and advance the development of single-chip DAS systems.

## 1. Introduction

Distributed acoustic sensing (DAS) technology has experienced rapid advancements in recent years, demonstrating great practical value in various fields. Although the fundamental concepts of DAS systems share certain similarities, there are distinct principles and implementations for lightwave phase demodulation and spatial division multiplexing. The phase-sensitive optical time domain reflectometer (ϕ-OTDR) is a prominent category of these methods, employing an optical frontend to transform the lightwave phase into measurable physical quantities, while utilizing the propagation time of light for spatial positioning.

DAS interrogation units (electronic and computing devices for demodulating the optical signal into strain or strain rate) are often implemented based on personal computers (PCs) and data acquisition (DAQ) daughtercards [1,2,3,4]. Some studies have focused on the issue of computational efficiency and employed graphics processing units (GPUs) [5,6,7] and field-programmable gate arrays (FPGAs) [8] as linear algebra accelerators. Despite the rich computational resources of modern computers, the real-time demodulation of the vast volume of signals generated by DAS remains a significant engineering challenge. Also, computer-based DAS systems exhibit certain limitations, such as large footprints and high power consumption.

The ϕ-OTDR system shares some fundamental principles with LiDARs [9] (light detection and ranging; pulsed operation, time-of-flight ranging) and radio communication systems [10] (heterodyne mixing, filtering), despite partially operating in the optical domain. Modern radio frequency (RF) devices, such as 5G modems and automotive radars, leverage very large-scale integration (VLSI) technologies, which combine analog, digital, and RF components on a single chip, making them compact, efficient, and inexpensive. The demodulation algorithms for communication and radar systems, having undergone years of development and being deeply optimized for digital hardware implementation, represent one of the key factors enabling their practical realization.

A key challenge in developing single-chip DAS interrogators lies in the design of hardware-efficient digital ϕ-OTDR algorithms and architectures, which necessitates a co-design approach between algorithmic development and hardware implementation. The design task must carefully consider the algorithm’s efficiency on hardware, as well as constraints related to bus bandwidth, logic resources, power consumption, and latency. Overcoming these hurdles would enable the development of compact, low-power DAS devices suitable for deployment in harsh, off-grid and remote environments, thereby contributing to applications from seismic research to next-generation infrastructures.

This article focuses on the development of hardware-efficient phase demodulation algorithms for heterodyne detection ϕ-OTDR systems. The rest of this paper is organized as follows: Section 2 introduces the heterodyne ϕ-OTDR frontend, the digital system architecture, and derives a signal model for simulation. Section 3 presents three phase demodulation methods (quadrature heterodyning, Hilbert transform, and wavelet transform) alongside their hardware co-design implementations, as well as our incremental DC-rejected phase unwrapper for robust phase-to-acoustic conversion. Section 4 details the experimental setup and benchmarking results, including local event positioning and fading noise suppression performance. Finally, Section 5 discusses comparative insights and practical implications.

## 2. System Setup and Signal Model

### 2.1. Optical Frontend

**Figure 1:**
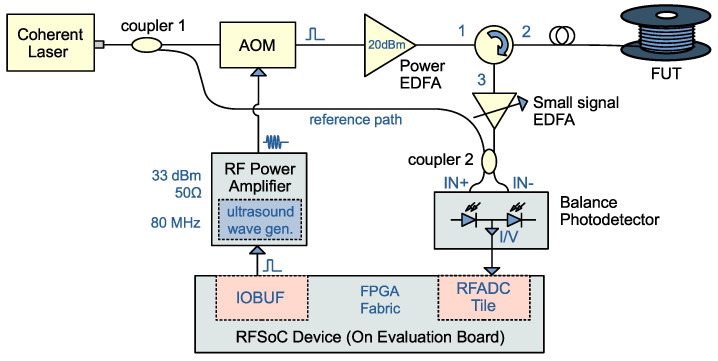
Optical setup of our targeted heterodyne ϕ-OTDR system. AOM: acousto-optic modulator; EDFA: erbium-doped optical fiber amplifiers; FUT: fiber under test; IOBUF: I/O buffer; RFADC: radio frequency analog-to-digital converter. Optical circulator: port 1 is input, port 2 is intermediate, port 3 is output.

Our study focuses on the well-established heterodyne detection optical arrangement, similar to studies [2,11,12,13]. The core principle involves generating an intermediate frequency beat signal through the interference between the signal light and a local oscillator light with a frequency offset, from which phase variations are extracted to detect external disturbances. The configuration is shown in Figure 1.

The highly coherent laser source (Guangyi Technology, Guilin, China) provides a continuous wave laser beam with 1550 nm wavelength, which is immediately divided into two paths. The probing path is modulated and amplified to create a pulse; the reference path is used as optical local oscillation. The acousto-optic modulator (AOM; Bonphot FAOM-1550, Suzhou, China) is driven by a specially designed RF power amplifier. The amplifier receives a gating signal. When asserted, a phase coherent 80 MHz sine wave is internally generated and amplified. The RF electrical current drives the piezoelectric material in the AOM, generating ultrasonic waves in the glass medium. In this condition, the first-order diffracted beam passes through the AOM, with the output lightwave frequency Doppler shifted by +80 MHz.

The pulsed probe travels through the fiber, generating Rayleigh backscattered (RBS) light from microscopic inhomogeneities. The RBS light retains the Doppler shift and accumulates phase changes from external disturbances. The RBS light, amplified by a forward pumped low-noise erbium-doped optical fiber amplifier (EDFA), is combined with the reference lightwave by a coupler before entering an AC-coupled balance photodetector, creating a very-high-frequency (VHF) band light beat, which carries the phase difference between the paths, encoding information about mechanical disturbances.

### 2.2. On-Chip Digital System

**Figure 2:**
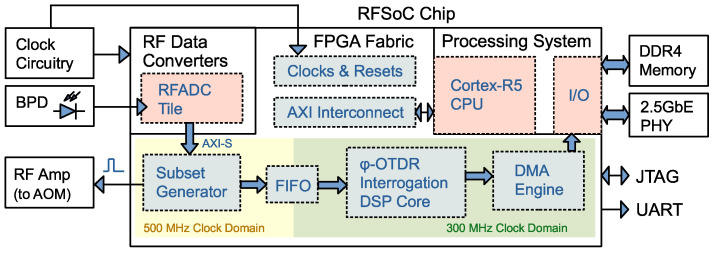
A simplified view of the ϕ-OTDR digital receiver structure. Parts that are less relevant to this study, such as the flash memory interface, analog front-end protection and debugging components, are not drawn in the figure. BPD: balance photodiode; RFADC: radio frequency analog-to-digital converter; FIFO: first-in first-out; DMA: direct memory access; JTAG: Joint Test Action Group; UART: universal asynchronous receiver/transmitter.

The basis of our digital ϕ-OTDR receiver is an RFSoC integrated circuit(Xilinx UltraScale+ XCZU47DR, sourced from Shanghai, China). Figure 2 gives a simplified view of the on-chip and off-chip hardware architecture.

The on-chip RF data converter (RFDC) is used to sample and quantize the light beat signal from the balanced photodetector; a Cortex-R5 CPU core is programmed to initialize the IP cores and clock peripherals, control the network interface, as well as handling interrupts from RFDC and the direct memory access (DMA) engine. All other digital hardware is implemented on the FPGA fabric.

ϕ-OTDR traces seen by RFDC can be viewed as a video signal with line synchronization but without frame synchronization, modulated into a frequency band, where each line corresponds to the pulse response of the sensing fiber at a specific time. The Subset Generator serves as a trigger control mechanism. It is a state machine that sends gating pulses to the AOM’s power amplifiers according to the required scan rate and gauge length. It also keeps track of the ϕ-OTDR trace sampling point index within the RFDC’s continuous data stream to establish line synchronization. The ϕ-OTDR Interrogation Core represents the hardware implementation of the algorithm described in this paper. This circuit takes a ϕ-OTDR trace as input and outputs the estimated instantaneous optical phase for each point on the trace. The DMA engine transfers the demodulated phase timeseries to the double data rate 4 synchronous dynamic random-access memory (DDR4 SDRAM) attached to the processing system (PS) dynamic random-access memory (DRAM) controller.

### 2.3. Signal Model for Received Traces

It has been reported that the strain-induced phase modulation of light in single-mode optical fiber, contributed by the changes in fiber length and refractive index, has a near-linear functional dependence [14]. Our model is formulated from lightwave phase modulation to the raw traces as recorded by ADC.

The optical system inherently exhibits both a stochastic initial phase and a gradual phase drift (induced by laser operation point drifting, thermal variations, etc.). Both effects can be modeled by a bandwidth-limited random walk process. The random walk is defined as the integration of a random variable of Gaussian distribution when considering the differential phase between sampling intervals.

(1) $$\Delta \phi_{0}\left[n\right]=L_{\mathrm{init}}\left\{\eta_{0}\left[n\right]\right\},\;\eta_{0}\left[n\right]∼N\left(0,G_{\mathrm{init}}^{2}\right)$$

where $L_{\mathrm{init}}$ represents a first-order Butterworth low-pass filter, with a cut-off frequency of $f_{\mathrm{init}}$, and $G_{\mathrm{init}}$ sets the gain for the initial phase random walk. Similarly, with lower bandwidth and gain settings, the differential phase drift is calculated along the probing time.

(2) $$\Delta \phi_{d}\left[m\right]=L_{\mathrm{drift}}\left\{\eta_{1}\left[m\right]\right\},\;\eta_{1}\left[n\right]∼N\left(0,G_{\mathrm{drift}}^{2}\right)$$

Combining the effects of the stochastic initial phase and drifting, the intrinsic differential phase matrix can be computed

(3) $$\Delta \phi_{\mathrm{intrins}}\left[n,m\right]=\Delta \phi_{0}\left[n\right]+\Delta \phi_{d}\left[m\right]$$

In the model, the sound wave is represented as an absolute phase time series. Window function terms are utilized to represent the acoustic wave irradiating to a specific position (or relatively uniformly distributed) along the optical fiber. Gain of the sound wave, as detected by ϕ-OTDR has a linear dependence on the gauge length. The sound amplitude in the model is defined as the case where the gauge length is equal to the pulse width. The acoustic contribution to the phase difference is:

(4) $$\Delta \phi_{\mathrm{sound}}\left[n,m\right]=\frac{1}{w_{\mathrm{pulse}}}\cdot s\left[m\right]\cdot w\left[n\right]$$

where s(m) is the acoustic timeseries (absolute phase, unit is radian), $w_{\mathrm{pulse}}$ is the gauge length (unit is the number of sampling points), and w(n) is the window function. In our study, the suppression method for fading was investigated by employing a uniformly distributed acoustic field, while the precision of localization was examined through a point source. Therefore, two window functions were utilized:

- For a uniform, homogeneous acoustic wave field:
  (5) $$w\left[n\right]=\left\{\begin{array}{cc} 1 & \mathrm{for}\;n\;\mathrm{within}\;\mathrm{wavefield}\;\mathrm{boundary},\\ 0 & \mathrm{for}\;\mathrm{others}. \end{array}\right.$$
- For a point source with energy concentrated near position μ and with a full-width-at-half-maximum (FWHM) of $w_{\mathrm{ps}}$:
  (6) $$w\left[n\right]=\exp\left(-\frac{{\left(n-\mu \right)}^{2}}{2\cdot \sigma^{2}}\right),\;\sigma =\frac{w_{\mathrm{ps}}}{2\sqrt{2\cdot \ln(2)}}\approx \frac{w_{\mathrm{ps}}}{2.355}$$

Combining the contributions of the intrinsic and sound-induced phase shifts, the differential phase at the *n*-th sampling point of the *m*-th trace is

(7) $$\Delta \phi \left[n,m\right]=\Delta \phi_{\mathrm{intrins}}\left[n,m\right]+\Delta \phi_{\mathrm{sound}}\left[n,m\right]$$

The absolute phase matrix becomes

(8) $$\phi \left[n,m\right]=\sum_{k=1}^{N}\Delta \phi \left[n,m\right]=\sum_{k=1}^{N}\left(\Delta \phi_{\mathrm{intrins}}\left[k,m\right]+\Delta \phi_{\mathrm{sound}}\left[k,m\right]\right)$$

Finally, the timeseries of the *m*-th trace as sampled by ADC is

(9) $$V\left[n,m\right]=c\left[n\right]\cdot \cos\left(2\pi \cdot f_{\mathrm{LO}}\cdot \frac{n}{f_{s}}+\phi \left[n,m\right]\right)+N_{s}\left[n,m\right]$$

where $f_{\mathrm{LO}}$ is the beat frequency (80 MHz for our configuration), $c_{n}$$f_{s}$ is the sampling rate of ADC, and $N_{s}$ is a random variable representing the additive white noise introduced in each link in the signal chain.

**Figure 3:**
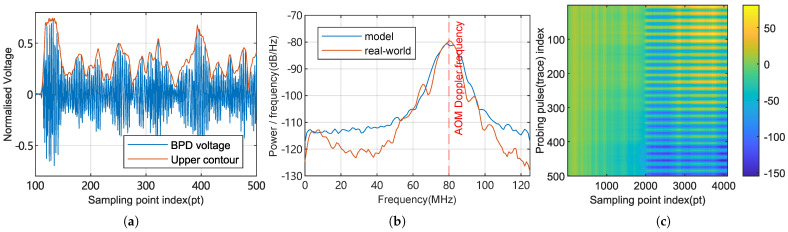
A simulated output of the signal model, with parameters set to typical values. (**a**) The first ϕ-OTDR trace as seen by BPD (fiber response after transmitting the probe pulse). Spatial distance corresponding to each sampling point is roughly 0.8 m. The contour is computed using the Hilbert transform. (**b**) The power spectral density estimation of a simulated and real-world measured ϕ-OTDR trace, computed using Welch’s method. (**c**) The absolute phase matrix visualized as an image.

Figure 3 provides simulated output of the signal model, where the parameters are tuned to statistically resemble our real-world ϕ-OTDR frontend in both the time and frequency domains.

## 3. Demodulation Algorithms

### 3.1. Phase Extraction

#### 3.1.1. The Quadrature Heterodyning Method

The heterodyne ϕ-OTDR optical path structure leverages the optical beat effect to down-convert the lightwave carrier to a VHF band intermediate frequency (IF) carrier in the optical domain. Following the optical-to-electrical conversion process, the resultant signal, in the form of a digital data stream, can be down-converted again in the digital domain to a zero intermediate frequency (ZIF), thereby reconstructing the baseband signal that accurately encodes the phase information of the RBS lightwave.

**Figure 4:**
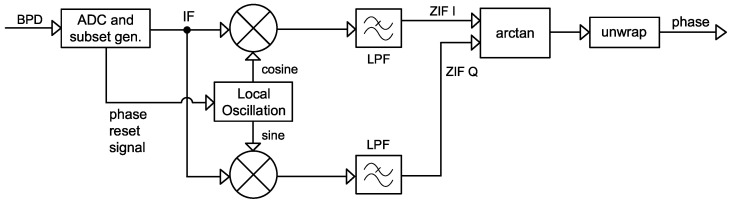
Block diagram for the quadrature heterodyning phase extraction method. LPF: low-pass filter; IF: intermediate frequency; ZIF: zero intermediate frequency; I: in-phase channel; Q: quadrature channel.

Figure 4 shows the signal processing flow of this method.

For a received trace length of *N* samples and an ADC sampling rate of $f_{s}$, define the discrete time vector and a pair of quadrature local oscillations:

(10) $$t\left[n\right]=\frac{n}{f_{s}},\;n=0,1,\ldots ,N-1\;$$

(11) $$\left\{\begin{array}{cc} {\mathrm{LO}}_{I}\left[n\right] & =G_{\mathrm{LO}}\cdot \cos\left(2\pi \cdot f_{\mathrm{LO}}\cdot t\left[n\right]\right)\\ {\mathrm{LO}}_{Q}\left[n\right] & =G_{\mathrm{LO}}\cdot \sin\left(2\pi \cdot f_{\mathrm{LO}}\cdot t\left[n\right]\right) \end{array}\right.$$

To translate IF to ZIF, for each trace m∈0,1,…,M, perform the mixing operation, then use a low-pass filter L to retain the signal near DC and filter out the high-order frequency terms in the mixing result.

(12) $$\left\{\begin{array}{cc} {\mathrm{ZIF}}_{I}^{\mathrm{filt}}\left[n,m\right] & =L_{\mathrm{ZIF}}\left\{{\mathrm{LO}}_{I}\left[n\right]\cdot V\left[n,m\right]\right\}\\ {\mathrm{ZIF}}_{Q}^{\mathrm{filt}}\left[n,m\right] & =L_{\mathrm{ZIF}}\left\{{\mathrm{LO}}_{Q}\left[n\right]\cdot V\left[n,m\right]\right\} \end{array}\right.$$

Then, the instantaneous phase can be computed using

(13) $$\phi_{\mathrm{inst}}^{\mathrm{HT}}\left[n,m\right]=\mathrm{atan}2\left({\mathrm{ZIF}}_{Q}^{\mathrm{filt}}\left[n,m\right],{\mathrm{ZIF}}_{I}^{\mathrm{filt}}\left[n,m\right]\right)\in \left(-\pi ,\pi \right]$$

This signal processing method largely resembles a ZIF communication receiver, and the approach can be efficiently implemented on the RFSoC’s digital system. The mixing of the real-valued optical beat into I/Q components can be achieved by the hardware datapath within the RFDC Tile without requiring additional logic resources. The most significant computational overhead lies in the ZIF filters. To ensure a linear phase response, which is important for maintaining the orthogonality of I/Q channels, we designed an 8-order finite impulse response (FIR) filter using the equiripple (Parks–McClellan) method, then converted the filter to a fixed-point precision pipelined version using the FIR compiler kernel. Two identical filters are instantiated to concurrently process the I and Q channel data. Similarly, the atan2 (arctan2) function can be computed through a fully pipelined coordinate rotation digital computer (CORDIC) kernel, achieving a throughput of one sample per clock cycle after the initial latency.

The final unwrapping stage corrects the discontinuities (±2π jumps) within phase series extracted from one trace. The goal is to compute

(14) $$\phi_{\mathrm{inst}}^{\mathrm{unwrap}}\left[n,m\right]=\phi_{\mathrm{inst}}\left[n,m\right]+2\pi \cdot K\left[n,m\right]$$

where K[n,m] is the integer number of phase wraps incremented/decremented when adjacent samples differ by more than π. To avoid the unnecessary computational complexity of directly computing 2π·K[n,m], the unwrapping algorithm can be performed incrementally as follows:

1. Compute phase difference: $\Delta \phi_{\mathrm{inst}}=\phi_{\mathrm{inst}}\left[n,m\right]-\phi_{\mathrm{inst}}\left[n-1,m\right]$.
2. Detect jumps ($\left|\Delta \phi_{\mathrm{inst}}>\pi \right|$) and determine correction step (±2π or 0).
3. Update cumulative correction.
4. Output $\phi_{\mathrm{inst}}^{\mathrm{unwrap}}\left[n,m\right]=\phi_{\mathrm{inst}}\left[n,m\right]+\mathrm{correction}\left[n,m\right]$.

This enables a pipelined, single-cycle implementation of the phase unwrapping stage.

Table 1 shows an estimation of the hardware resources required to implement the quadrature heterodyning ZIF demodulator.

#### 3.1.2. Hilbert Transform Method

In addressing the problem of phase extraction, the Hilbert transform(HT) is utilized as a particularly suitable mathematical tool when considering IF as a narrowband signal with composite amplitude and phase modulation (AM-PM). This approach operates directly in the time domain, therefore eliminating the complexity of additional frequency conversion and filtering stages.

**Figure 5:**
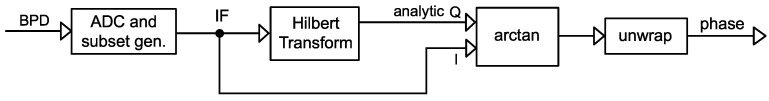
Block diagram for the Hilbert transform phase extraction method.

Figure 5 shows the signal processing flow of this method.

For the *m*-th received trace, the IF is converted into a complex-valued analytical signal A[n,m] using the Hilbert transform:

(15) A[n,m]=V[n,m]+j·H{V[n,m]}

where $j=\sqrt{-1}$. Then, the instantaneous phase is

(16) ϕinstZIF[n,m]=arg(A[n,m])=atan2(Im{A[n,m]},Re{A[n,m]})∈(−π,π]

Additionally, the instantaneous amplitude of the IF, i.e., the envelope, can also be calculated from the analytical signal:

(17) cinstZIF[n,m]=|A[n,m]|=Re{A[n,m]}2+Im{A[n,m]}2

The Hilbert transform applies a $\frac{\pi }{2}$ phase shift to all positive frequency components of a signal; our digital implementation approximates it with a finite-length FIR filter, which mimics the HT’s phase behavior near the IF frequency band. This avoids the infinite computation of HT’s ideal analog definition. A 30-order FIR Hilbert transformer with passband covering the IF bandwidth is utilized for this operation. Similarly to the IF filter in the quadrature heterodyning method, the Hilbert transformer can be implemented fully pipelined using the FIR compiler kernel. The hardware for the arctangent calculation and unwrapping stages is the same as in the quadrature heterodyning method, as described in Section 3.1.1.

Table 2 shows an estimation of the hardware resources required to implement the quadrature heterodyning ZIF demodulator.

#### 3.1.3. Complex Wavelet Transform Method

Interference fading is a phenomenon in ϕ-OTDR where coherent RBS lightwaves interfere destructively, leading to localized signal nulls (fades). This occurs due to the random distribution of scattering centers in the fiber. When destructive interference dominates, the detected signal at specific spatial drops significantly, creating “dead zones”. Various approaches have been investigated to mitigate signal-to-noise ratio (SNR) degradation caused by fading, with some achieving complete elimination of the associated sensing blind spots. The solutions encompass multiple techniques, including the following: implementing frequency hopping for laser sources [15], employing dual-laser configurations [16], applying linear frequency-sweep modulations [17,18], utilizing multiple probing frequencies [19], and fully exploiting the frequency components of rectangular pulses [20].

**Figure 6:**
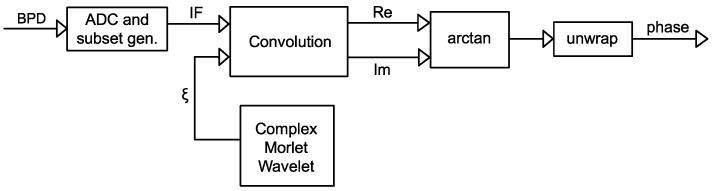
Block diagram for the complex Morlet wavelet transform phase extraction method. ξ: the wavelet; Re: real part of complex numbers; Im: imaginary part of complex numbers.

The optical beat is a narrowband, phase-modulated signal. To extract its instantaneous phase, the signal can be convolved with a complex wavelet. At each time point, the result of the convolution is a complex coefficient, and the phase at that particular point is represented by the angle of this coefficient. The Morlet wavelet is essentially a Gaussian-windowed complex sinusoid oscillation. The Gaussian window ensures the wavelet decays rapidly to near-zero away from the center, enabling localized time analysis; the sinusoid makes the wavelet sensitive to specific frequency—when convolved with a signal, it resonates with certain frequency components, amplifying their contribution. The real and imaginary parts of the complex Morlet wavelet form a quadrature pair (sine and cosine), allowing direct computation of the instantaneous phase. By selecting the bandwidth of the wavelet (number of oscillation cycles), the time/frequency resolution can be balanced. Higher cycles provide good phase estimation but poor transients, while lower cycles provide better time resolution but noisier phase output. These unique properties are particularly useful for demodulating ϕ-OTDR IF with fading spots, as local fading can be suppressed because the wavelet resonates with the signal in its neighborhood. Figure 6 shows the signal processing flow of this method.

To save memory, a suitable truncation time range is selected to generate a complex wavelet discrete sequence of finite length. The fast decay characteristic of the Gaussian window makes the error after truncation negligible. A symmetric time window is defined around t=0, with discrete steps of $\frac{1}{f_{s}}$, spanning across $\pm \frac{N_{\mathrm{cycles}}}{2\cdot f_{\mathrm{LO}}}$

(18) $$t\left[i\right]=-\frac{N_{\mathrm{cycles}}}{2\cdot f_{\mathrm{LO}}},\;i=0,1,\ldots ,I-1$$

where $I=N_{\mathrm{cycles}}\cdot \frac{f_{s}}{f_{\mathrm{LO}}}+1$ is the number of time points in the wavelet. This ensures the wavelet spans exactly $N_{\mathrm{cycles}}$ cycles at center frequency $f_{\mathrm{LO}}$.

A discrete-time version of the complex Morlet wavelet is computed by multiplying a complex exponential and a Gaussian window:

(19) $$\xi \left[i\right]=\exp\left(2\pi \cdot j\cdot f_{\mathrm{LO}}\cdot t\left[i\right]\right)\cdot \exp\left(-\frac{t{\left[i\right]}^{2}}{2\cdot \sigma^{2}}\right)$$

in which the standard deviation of the window is

(20) $$\sigma =\frac{N_{\mathrm{cycles}}}{2\pi \cdot f_{\mathrm{LO}}}$$

**Figure 7:**
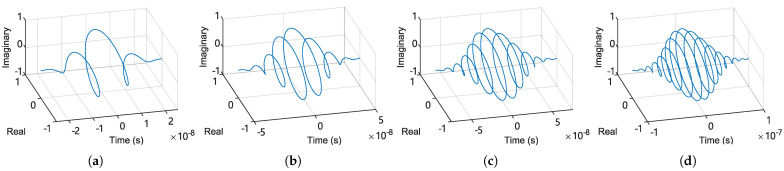
Computed complex Morlet wavelet (continuous time) plotted on complex axes, with four typical settings for the number of periods: (**a**) $N_{\mathrm{cycles}}=4$. (**b**) $N_{\mathrm{cycles}}=8$. (**c**) $N_{\mathrm{cycles}}=12$. (**d**) $N_{\mathrm{cycles}}=16$.

Figure 7 shows four typical examples of computed wavelets, with $N_{\mathrm{cycle}}$ set to 4, 8, 12, and 16. With the wavelet ready, discrete convolution for each trace m∈0,1,...,M can then be computed to obtain the complex wavelet coefficients:

(21) $$B\left[n,m\right]=\sum_{i=0}^{I-1}V\left[n+\left\lfloor \frac{I}{2}\right\rfloor -i,m\right]\cdot \xi \left[i\right]$$

where $\left\lfloor \frac{I}{2}\right\rfloor$ centres the wavelet at t=0.

The instantaneous phase can be computed using an arctangent operation:

(22) ϕinstWT[n,m]=arg(B[n,m])=atan2(Im{B[n,m]},Re{B[n,m]})∈(−π,π]

Compared with the HT method, the wavelet transform method introduces an additional parameter to balance the position accuracy and frequency accuracy. When implementing a digital system, the sampling rate, IF frequency, and wavelet cycle number parameters must be considered comprehensively to achieve an efficient and effective hardware implementation. In our system, with 80 MHz AOM Doppler shift and 250 MSa/s ADC data rate, when the number of wavelet cycles is 8–16, the corresponding discrete complex wavelet sample points are 26–51, at which point, convolution in the time domain can be efficiently completed using a standard FIR technique. Also, the wavelet cycle number of 8–16 is also a reasonable range that effectively balances interference fading suppression and positioning performance, as discussed in Section 4.2.

A complex wavelet can be pre-computed and quantized on the host computer and then embedded into the block read-only memories (ROMs) in the FPGA. The real and imaginary channels of wavelet convolution can be implemented fully pipelined by two identical FIR kernels each with 26–51 taps. The hardware for the arctangent calculation and unwrapping stages is the same as in the quadrature heterodyning method, as described in Section 3.1.1.

Table 3 shows an estimation of the hardware resources required to implement the complex wavelet transform demodulator.

### 3.2. Incremental DC-Rejected Phase Unwrapper

To obtain a natural acoustic timeseries suitable for playback or further signal analysis, the demodulated differential phase must undergo the following two post-processing steps: (1) phase unwrapping, which resolves 2π ambiguity to generate a continuous waveform free from discontinuities; and (2) high-pass filtering, which removes the DC and low-frequency components induced by thermal effects and laser operating point drifts, yielding an acoustic waveform centered at zero. However, in most practical DAS applications, where the system must maintain continuous recording over long periods, the conventional two-step post-processing approach may exhibit numerical instabilities—if the unwrapped differential phase undergoes fast and/or multiple rotations in the complex plane, it can lead to unwrapping errors, or in extreme cases, the unwrapped values may exceed the upper or lower bounds of the digitally stored variables, resulting in overflow issues.

**Figure 8:**
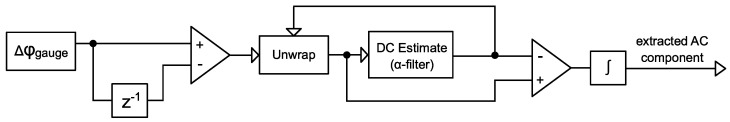
Block diagram for the incremental DC-rejected phase unwrapper (IDRPU) method. Unwrapping and filtering are combined in a single pass and numerical overflow is prevented.

An incremental DC-rejected phase unwrapper (IDRPU) method was proposed to extract the AC components from the wrapped phase signals while simultaneously tracking and suppressing the DC trends to prevent numerical overflow. The IDRPU method’s processing flow is shown in Figure 8.

For position *n* in the *m*-th-received ϕ-OTDR trace, a gauge length is applied first. This is performed by unwrapping the phase along the direction in which a single trace is sampled using a standard technique (adjust only by adding 2kπ), and then computing the phase difference between the two points.

(23) $$\Delta \phi_{\mathrm{gauge}}\left[n,m\right]=\phi_{\mathrm{inst}}^{\mathrm{unwrap}}\left[n+\frac{\mathrm{GL}}{2},m\right]-\phi_{\mathrm{inst}}^{\mathrm{unwrap}}\left[n-\frac{\mathrm{GL}}{2},m\right]$$

where GL is the gauge length (even number of sampling points).

The IDRPU method is used to recover the acoustic timeseries, starting from initialization of the state variables $\hat{A}\left[n,0\right]$ and $\hat{A}\left[n,0\right]$, which represent the AC and DC estimates within the algorithm, respectively.

(24) $$\hat{A}\left[n,0\right]=0,\;\hat{D}\left[n,0\right]=0$$

First, compute the difference between adjacent received traces:

(25) $$\Delta \phi_{\mathrm{diff}}\left[n,m\right]=\Delta \phi_{\mathrm{gauge}}\left[n,m\right]-\Delta \phi_{\mathrm{gauge}}\left[n,m-1\right]$$

At this stage, adjust $\Delta \phi_{\mathrm{diff}}\left[n,m\right]$ by adding 2k[n,m]·π:

(26) $$\Delta \phi_{\mathrm{diff}}^{\mathrm{unwrap}}\left[n,m\right]=\Delta \phi_{\mathrm{diff}}\left[n,m\right]+2k\left[n,m\right]\cdot \pi$$

(27) $$k\left[n,m\right]=\left\lfloor \frac{\hat{D}\left[n,m-1\right]-\Delta \phi_{\mathrm{diff}}\left[n,m\right]}{2\pi }\right\rceil$$

Then, the DC estimation is updated using a single-parameter infinite impulse response (IIR) filter.

(28) $$\hat{D}\left[n,m\right]=\alpha \cdot \hat{D}\left[n,m-1\right]+\left(1-\alpha \right)\cdot \Delta \phi_{\mathrm{diff}}^{\mathrm{unwrap}}\left[n,m\right]$$

where the parameter α determines the DC estimator’s cut-off frequency.

Finally, accumulate the deviation from DC estimation to obtain the AC component:

(29) $$\hat{A}\left[n,m\right]=\hat{A}\left[n,m-1\right]+\left(\Delta \phi_{\mathrm{diff}}^{\mathrm{unwrap}}\left[n,m\right]-\hat{D}\left[n,m\right]\right)$$

The variable $\hat{A}\left[n,m\right]$ denotes the audio signal at position *n*; however, the signal is expressed in radians, which is an unconventional unit for an audio signal. Given the near-linear strain-induced phase modulation characteristic, this signal can be amplified and subsequently used for audio playback or further processing.

## 4. Experimental Measurements

### 4.1. Data Acquisition Setup

Numerical simulations and a real-world system were employed in evaluating the relative performance of the investigated demodulation schemes. Considering the typical application scenarios of DAS in distributed acoustic field measurement and positioning of acoustic events, two configurations were designed:

1. The entire sensing fiber was exposed to a uniform acoustic field, where all the sensing points received test acoustic waves with near uniform amplitude. This configuration was employed to evaluate the noise characteristics of the demodulation schemes, particularly the impact of interferometric fading phenomena on signal quality. The phase of the sound wave received by this setup is not uniform, so the phase must be compensated in the step of calculating the SNR.
2. The sensing fiber was placed in a quiet environment, while a narrow segment along the fiber received the testing acoustic wave. This configuration was utilized to evaluate the localization accuracy of the demodulation schemes.

**Figure 9:**
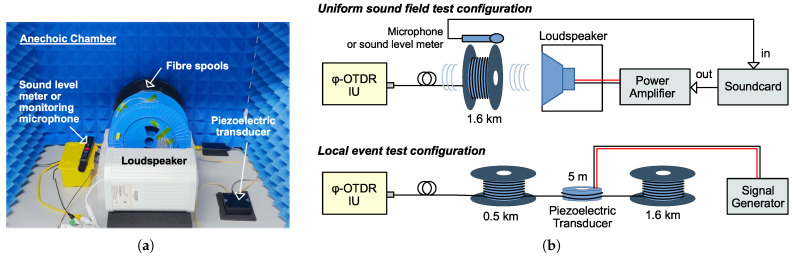
Experimental setup for acquiring ϕ-OTDR trace data. Two configurations are used to separately evaluate the positioning capability and noise characteristics. (**a**) Photograph of the setup. (**b**) Block diagrams illustrating the two configurations designed for capturing uniform acoustic fields and localized events.

Figure 9 shows a photograph of the experimental setup and block diagrams of the two configurations.

### 4.2. Performance Measurement of Phase Extraction Methods

In Section 3.1, the methodology for extracting phase information from the acquired optical beat signal is introduced. Though quadrature heterodyning ZIF, Hilbert transform, and wavelet transform methods serve analogous roles in the ϕ-OTDR receiver system, their inherent properties and characteristics vary due to differences in their mathematical principles. This section evaluates the methods based on two key performance metrics: positioning accuracy and interferometric fading suppression capability. This provides insights to guide the design of digital systems and inform the selection of the most suitable algorithm for addressing specific practical problems. The parameters in Table 4 define a virtual ϕ-OTDR receiver which has a resemblance to our hardware setup.

#### 4.2.1. Local Acoustic Event (Positioning Task)

A simulation is designed to evaluate the capabilities of three phase extraction methods in positioning local events. A point-like acoustic source with a sharp spatial profile is added into the simulation model and optical beat signals (ϕ-OTDR traces) are generated with additional parameters in Table 5. The traces are subsequently demodulated using three methods.

The demodulated differential phase time series is fitted with 1 kHz sinusoidal functions to extract the amplitude at each spatial position along the optical fiber. The relationship between the amplitude and the spatial position (i.e., the sampling point index) is plotted to visualize the spatial distribution of the acoustic response.

**Figure 10:**
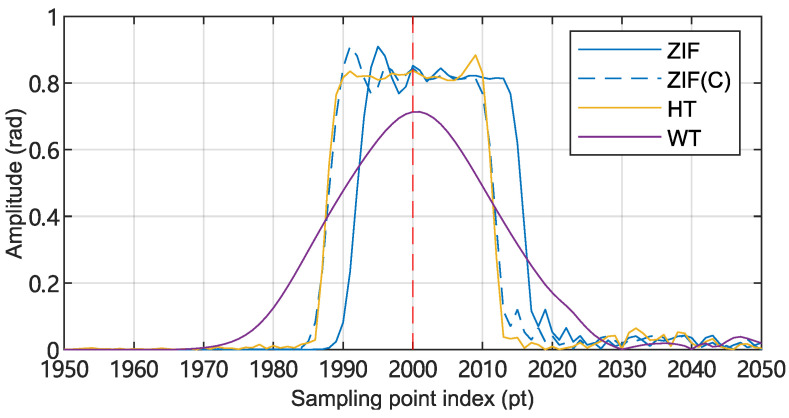
Positioning characteristics of the three phase extraction methods. A virtual 1 kHz point source is placed at position n=2000 (indicated by the red dashed line). The figure plots the relationship between the demodulated signal’s amplitude and position (i.e., sampling point index *n*). ZIF: quadrature heterodyning zero intermediate frequency (ZIF(C) for the shift-compensated result); HT: Hilbert transform; WT: wavelet transform.

An analysis of Figure 10 reveals that:

- HT method: an accurate and statistically confident positioning result can be inferred. The HT method successfully identifies the acoustic source within a region centered at position 2000, with a spatial extent corresponding to one gauge length.
- Quadrature heterodyning ZIF method: similar to HT, a highly confident positioning result (within a spatial range of one gauge length) is inferred. However, the estimated central position of the acoustic source exhibits a deviation of four sampling points toward the distal end of the optical fiber. The shift is caused by the group delay of the FIR ZIF low-pass filter in the down-conversion stage, and the effect can be compensated by shifting the phase sequence by four sample points towards the beginning of the fiber (shown in Figure 10 as a blue dashed curve).
- WT method: a result characterized by high accuracy yet elevated statistical dispersion (i.e., positional uncertainty) can be inferred. This is because the wavelet transform achieves a trade-off between temporal and frequency resolution by selecting the width of the analysis window. A broader temporal window is selected for accurate estimation of the signal’s phase information, and this comes at the expense of reduced precision in temporal localization.

For the wavelet transform method, the position accuracy is related to the size of the wavelet window. To evaluate this relationship, the demodulation was repeated using different numbers of complex Morlet wavelet cycles $N_{\mathrm{cycles}}$ and the FWHM of the resulting signal amplitude was analyzed.

**Figure 11:**
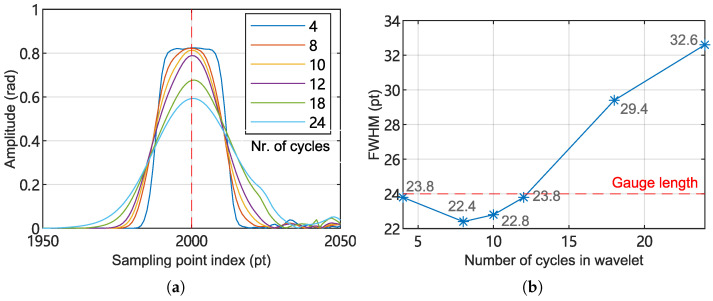
Positioning performance of WT method tested under varying wavelet cycle numbers. (**a**) Acoustic signal amplitude as a function of position (sampling point index). (**b**) FWHM of signal intensity curves as a function of wavelet cycle numbers.

Figure 11 presents the results of localization performance under varying wavelet cycle numbers. It can be observed that when the wavelet cycle number is below 12, the FWHM of the acoustic intensity distribution remains within one gauge length, demonstrating positioning capabilities comparable to those achieved by the ZIF and HT methods.

**Figure 12:**
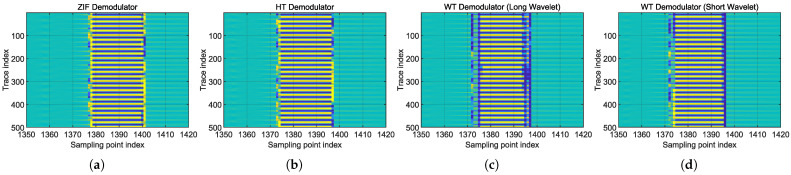
Phase timeseries “waterfall” diagrams of real-world piezoelectric vibration measurement, using all demodulation schemes. (**a**) Quadrature heterodyning ZIF method. (**b**) HT method. (**c**) WT method with $N_{\mathrm{cycles}}=16$. (**d**) WT method with $N_{\mathrm{cycles}}=8$.

The three demodulation schemes were also applied to the real-world data (introduced in Section 4.1) to validate their respective characteristics. Figure 12 presents the demodulation results in the form of phase timeseries “waterfall” diagrams, as commonly used for DAS data visualization. The group delay phenomenon in the quadrature heterodyne ZIF method can be observed, along with the differences in positioning uncertainty between long and short wavelets. When employing long wavelet ($N_{\mathrm{cycles}}=16$), the vibration event could still be localized within one gauge length, but the event boundaries appeared relatively blurred compared to other methods. In contrast, the use of short wavelet ($N_{\mathrm{cycles}}=8$) yielded localization results comparable to the HT method, demonstrating accurate positioning with clear boundaries.

#### 4.2.2. Acoustic Field Measurement (Fading Noise Suppression)

**Figure 13:**
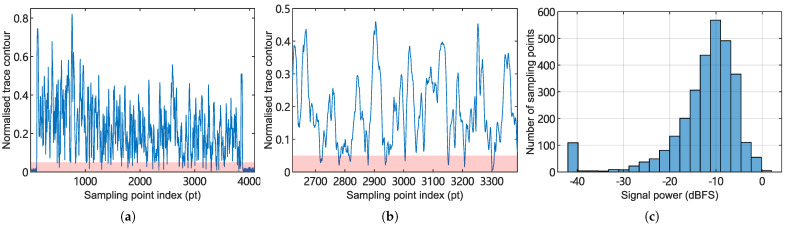
A real-world measured RBS intensity trace showing the fading effect. The trace is obtained by computing the envelope of the beat frequency signal using the Hilbert transform method. (**a**) RBS intensity as a function of the sample point index (i.e., position along the fiber). (**b**) A local view of the waveform in (**a**). (**c**) Statistical distribution of light intensity. The first bin (−40 dBFS) indicates positions outside the measured fiber, which can be excluded from the statistics.

The fading issue associated with ϕ-OTDR was discovered and attempted to be resolved. A fading pattern measured from a real world setup is plotted in Figure 13. Experiments are designed and conducted using both simulated and measured traces to evaluate the demodulators’ robustness against interferometric fading.

Even though the interferometric fading in the real world is chaotic and random, and the condition of constant optical signal SNR almost never occurs, this special case can still be simulated to evaluate the relationship between optical SNR and acoustic SNR.

The simulation model is configured such that the entire sensing fiber is exposed to a uniform weak acoustic field. Instead of adding random fading, the noise term in Equation (9) is adjusted to create optical beat signals with constant SNR (Table 6).

**Figure 14:**
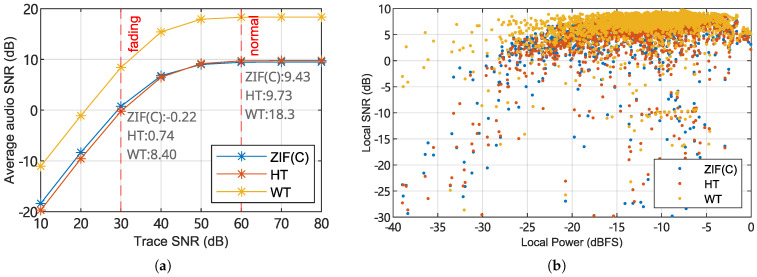
(**a**) Three demodulation schemes’ output audio signal SNR as functions of ϕ-OTDR trace SNR, computed using simulated controlled conditions, where the fading effect is uniform and has no fluctuations. (**b**) Scatter plot of the relationship between the short-term sound signal SNR and the instantaneous light beat SNR, using real-world sampled data and 3 studied demodulation schemes. The interferometric fading suppression effect of the WT method can be observed.

Figure 14a shows the test results. Under the condition of limited optical beat signal SNR, the quadrature heterodyning ZIF method and HT method produced similar audio signal SNR. The audio SNR of the WT method is globally higher than that of the other two methods. In the fading condition, the WT method produced around +8dB SNR gain compared to the HT and ZIF methods. This shows that for the same standard of acceptable audio quality, the WT method has a stronger tolerance to interferometric fading.

Unlike the simulated case, the real-world ϕ-OTDR signal has complex interferometric fading fringes and intrinsic drift effects, while the optoelectronic receiver has a constant noise floor, so a wide range of optical signal SNR conditions will occur at every position of every received trace. Real-world ϕ-OTDR traces were demodulated using the same methods and parameters to evaluate the interferometric fading suppression effects.

The data acquisition process is conducted with a spool of 1.6 km of fiber attached, and the speaker emitting a 1 kHz sinusoid sound wave. Because the interferometric fading pattern is time-varying, the recorded trace sequence is split into segments of 0.5 s in length, and the fading pattern and SNR in each segment are analyzed separately. Also, because the size of the fiber spool is comparable to the wavelength of the sound wave, the phase of the sound wave received at different positions on the fiber spool may be different. The SNR is calculated by restoring the 1kHz clean signal through the least squares method to calculate the signal power and noise power.

**Figure 15:**
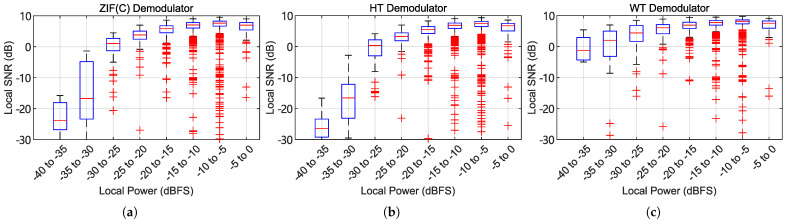
Box plot of the relationship between the short-term sound signal SNR and the instantaneous light beat SNR, using real-world sampled data and all 3 demodulation schemes. (**a**) The quadrature heterodyning ZIF method with group delay compensated. (**b**) The Hilbert transform method. (**c**) The wavelet transform method.

Figure 14b and Figure 15 and Table 7 show the statistics of the instantaneous signal power of the traces versus the short-term audio SNR. The overall trend of the output SNR is similar to that in the simulation experiment. At the locations where fading occurs (instantaneous signal strength dropping below −25 dBFS), the WT method maintains usable audio quality, confirming the WT method’s ability to suppress interferometric fading. Moreover, unlike the simulated constant-SNR special case, the fading zones in real data have limited widths, which means that the wavelet convolution process can utilize the context of the local waveform to estimate the phase of the fading location at the expense of spatial resolution. Therefore, WT shows a better suppression effect under actual fading conditions than in simulation experiments.

**Figure 16:**
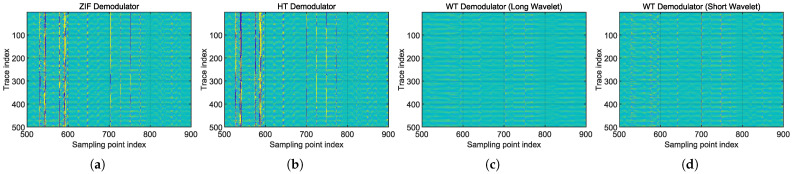
Phase timeseries “waterfall” diagrams of real-world weak acoustic field measurement, using all demodulation schemes. The random stripes running vertically are the SNR drops caused by interferometric fading. The diagrams were cropped so that local areas with strong and concentrated noise are shown. (**a**) Quadrature heterodyning ZIF method. (**b**) HT method. (**c**) WT method with $N_{\mathrm{cycles}}=16$. (**d**) WT method with $N_{\mathrm{cycles}}=8$.

The phase time-series “waterfall” diagrams are presented in Figure 16. It can be verified that the WT method effectively mitigates locally occurring interferometric fading, thereby eliminating the presence of positions with very low SNRs in the demodulated phase time-series.

## 5. Discussion

### 5.1. Comparison of Demodulation Schemes

The experimental results presented in Section 4 provide insights into the performance and characteristics of the studied demodulation schemes. Here, they are compared from the perspectives of performance characteristics and resource usage.

The quadrature heterodyning ZIF and HT methods demonstrated similar positioning performance and noise characteristics in our tests. Both methods were capable of accurately localizing acoustic events within the specified gauge length. The ZIF method required additional consideration for group delay compensation for its ZIF filters, but this can be easily resolved by shifting the demodulated phase series. We propose that the HT method can serve as a simplified implementation of the conventional ZIF approach while achieving virtually equivalent performance. This suggests that these approaches are well suited for resource-constrained FPGA implementations. The HT method, with its straightforward implementation and lower resource consumption, emerges as a practical choice for real-time, large-scale DAS systems.

The WT method introduces an additional parameter (the number of wavelet cycles), enabling flexible configuration to trade positioning accuracy for higher audio SNR, or vice versa. Our tests revealed that for an AOM Doppler frequency of 80 MHz and ADC sampling rate of 250 MSa/s, using eight wavelet cycles resulted in significantly improved interference fading suppression without substantially increasing the ambiguity of acoustic event boundaries. Performance of the WT method in balancing positional and phase precision, along with its ability to effectively suppress interferometric fading, highlights its potential for high-fidelity or blind-spot-free applications.

When considering hardware implementation, all three methods can be efficiently realized using digital logic resources on FPGAs, achieving a throughput of one sample per clock cycle through fully pipelined designs. Within the RFSoC architecture, most computations can be executed with digital signal processing (DSP) slices. The WT method typically requires approximately two to four times more DSP slices compared to the other two methods, depending on the complex wavelet length, but its robustness and fading suppression ability justify the adoption. The higher hardware resource consumption of WT underscores the need for consideration of system requirements and available resources. This trade-off emphasizes the importance of algorithm-hardware co-design in optimizing FPGA-based solutions.

### 5.2. Implications of the Findings

1. This study presents reference designs of the digital receiver for heterodyne detection ϕ-OTDR systems, featuring fully pipelined real-time processing datapaths.
2. This study gives comparative evaluations of three methodologies for processing optical beat signals into phase sequences: (1) a baseband reconstruction approach through down-conversion to zero intermediate frequency, (2) an analytic signal generation technique employing the Hilbert transform, and (3) an alternative analytic signal generation method utilizing complex wavelet transform. The study also provides algorithm-hardware co-design insights, including resource utilization estimates for RFSoC and standard FPGA platforms.
3. This study proposes a computationally efficient phase unwrapping method that integrates high-pass filtering, eliminating DC drift-induced numerical overflow while reducing processing steps.
4. This study conducts systematic performance comparisons using simulated data and field-measured ϕ-OTDR signals. Acoustic event positioning accuracy and interference fading noise suppression capabilities are evaluated and discussed. The study also offers trade-off analysis between computational efficiency and demodulation performance, aiding the selection of optimal phase extraction methods for different DAS applications.

### 5.3. Limitations of the Study

1. The optical beat signal model presented in this study is specifically designed to generate signals with controlled conditions for the experimental validation, rather than constituting a complete and exhaustive signal representation. For instance, the model assumes stationary interference fading patterns within short time windows, and the intrinsic phase drift is approximated statistically without accounting for real-world physical mechanisms.
2. The IDRPU algorithm presented in this work incorporates a single-parameter DC estimator, which functionally equates to a first-order IIR high-pass filter. While this implementation is sufficiently effective for general-purpose acoustic sensing, precise seismic measurements necessitate careful consideration of the filter’s magnitude and phase frequency characteristics. In such cases, the design of higher-order filters may be required. This design approach stems from hardware efficiency considerations, as the IDRPU module needs to be instantiated multiple times depending on specific system configurations.
3. The paper demonstrates the ability of wavelet convolution to suppress interference fading noise. This is a simple method that does not require increasing the complexity of the optical path or the modulation signal. If a very narrow audio event happens to occur at a fading centre, phase information at that point is still lost or corrupted, meaning that the sensing system still has blind spots. More complex hardware is still necessary if true blind-spot-free sensing is required.

### 5.4. Perspective and Final Thoughts

We firmly believe that hardware-algorithm co-design is of paramount importance for advancing distributed fiber-optic sensing systems. Optical multiplexing principles allow the scaling of sensor arrays to a new level, but data generated by OTDR (optical time domain reflectometry), OFDR (optical frequency domain reflectometry), FBG (fiber Bragg grating) arrays, etc., demand efficient real-time processing at the hardware level, making computational efficiency as critical to fiber sensing as it has been for transformative technologies like computer graphics, digital communications, and precision measurement. Just as these fields achieved breakthroughs through optimized hardware, the future of fiber-optic sensing lies in developing domain-specific architectures that jointly optimize physical-layer performance and computational overhead. Our work demonstrates how strategic co-design—from demodulation algorithms to FPGA implementations—can simultaneously enhance noise resilience, reduce latency, and lower potential cost. May this philosophy guide future research to unlock the full potential of distributed acoustic sensing for more innovative applications.

## Figures and Tables

**Table 1:** Estimation of hardware utilization for a quadrature heterodyning ZIF demodulator.

Stage	Components	Resources
Digital mixers ^1^	2 DDS cores for sine and cosine	2 Block RAMs
	Mixers for I/Q channels	2 DSP slices
ZIF filters	2 FIR cores	18 DSP slices
	Coefficient storage	~0.3 kbits Distributed RAM
	Control logic	~200 LUTs and FFs
Phase angle	Pipelined CORDIC core (16 iterations)	~1000 LUTs and FFs
	Pipelined unwrapper	~300 LUTs and ~400 FFs

RAM: random access memory; DSP: digital signal processing; LUT: look-up table; FF: flip-flop. ^1^ For RFSoC device, this is built into the RFDC tile so logic resources for this stage can be saved.

**Table 2:** Estimation of hardware utilization for an HT demodulator.

Stage	Components	Resources
Hilbert transformer	Antisymmetric FIR core	15 DSP slices
	Coefficient storage	~0.2 kbits Distributed RAM
	Control logic	~200 LUTs and FFs
	Delay line for real part	256 FFs
Phase angle	Pipelined CORDIC core (16 iterations)	~1000 LUTs and FFs
	Pipelined unwrapper	~300 LUTs and ~400 FFs

**Table 3:** Estimation of hardware utilization for a complex wavelet transform demodulator.

Stage	Components	Resources
Wavelet convolution	2 FIR cores	Up to 104 DSP slices
	Coefficient storage	~1.6 kbits Distributed RAM
	Control logic	~200 LUTs and FFs
Phase angle	Pipelined CORDIC core (16 iterations)	~1000 LUTs and FFs
	Pipelined unwrapper	~300 LUTs and ~400 FFs

**Table 4:** Parameters for phase extractors performance evaluation.

Type	Parameter	Value
Receiver configurations	ADC sampling clock speed	250 MSa/s
	Probe rate	20 kPulse/s
	Probe pulse width	100 ns (25 Sa)
Intrinsic fluctuations	$L_{\mathrm{init}}$ bandwidth ^1^	40 MHz
	$L_{\mathrm{drift}}$ bandwidth ^2^	5 Hz

^1, 2^ As defined in Equations (1) and (2).

**Table 5:** Additional parameters for positioning accuracy measurement.

Type	Parameter	Value
Receiver configurations	Gauge length	24 Sa
	$N_{\mathrm{cycles}}$ of wavelet ^1^	16
Intrinsic fluctuations	$G_{\mathrm{init}}$ gain ^2^	0.5
	$G_{\mathrm{drift}}$ gain ^3^	0.1
	BPD noise gain	$5\times 10^{-3}$
Acoustic source	FWHM ($w_{\mathrm{ps}}$)	2 Sa
	Peak amplitude	10 rad
Interferometric fading	No fading effects	

^1^ As defined in Equation (18). ^2, 3^ As defined in Equations (1) and (2). These are very small phase fluctuations compared to real-world situations.

**Table 6:** Parameters for constant trace SNR special case.

Type	Parameter	Value
Receiver configurations	Gauge length	24 Sa
	$N_{\mathrm{cycles}}$ of wavelet	8
Intrinsic fluctuations	$G_{\mathrm{init}}$	0
	$G_{\mathrm{drift}}$	0
	BPD noise gain	$5\times 10^{-3}$
Acoustic source	FWHM ($w_{\mathrm{ps}}$)	*∞*
	Peak amplitude	$50\times 10^{-3}$ rad

**Table 7:** Median SNRs of demodulators with all local power.

Range of local power (dBFS)	−40–−35	−35–−30	−30–−25	−25–−20	−20–−15	−15–−10	−10–−5
ZIF method’s median SNR (dB)	−23.78	−16.7	1.06	3.79	5.82	7.04	7.6
HT method’s median SNR (dB)	−26.44	−16.55	0.37	3.29	5.48	6.88	7.41
SNR gain of HT over ZIF (dB)	−2.66	+0.15	−0.69	−0.5	−0.34	−0.16	−0.19
WT method’s median SNR (dB)	−1.31	2.03	4.41	6.07	6.91	7.74	8.13
SNR gain of WT over ZIF (dB)	+22.47	+18.73	+3.35	+2.28	+1.09	+0.7	+0.53

## Data Availability

The data that support the findings of this study are available from the corresponding author upon reasonable request.

## Abbreviations

The following abbreviations are used in this manuscript:

ϕ-OTDR	Phase-sensitive optical time domain reflectometry
ADC	Analog-to-digital converter
AOM	Acousto-optic modulator
BPD	Balanced photodetector
CORDIC	Coordinate rotation digital computer
DAC	Digital-to-analog converter
DAQ	Data acquisition
DAS	Distributed acoustic sensing
DDR4 SDRAM	Double data rate 4 synchronous dynamic random-access memory
DMA	Direct memory access
DRAM	Dynamic random-access memory
DSP	Digital signal processing
EDFA	Erbium-doped optical fiber amplifiers
FBG	Fiber Bragg grating
FIFO	First-in first-out
FIR	Finite impulse response
FPGA	Field-programmable gate arrays
FUT	Fiber under test
FWHM	Full-width-at-half-maximum
GPU	Graphics processing unit
HT	Hilbert transform
I/Q	In-phase/quadrature
IF	Intermediate frequency
IIR	Infinite impulse response
IOBUF	Input/output buffer
JTAG	Joint Test Action Group
LiDAR	Light detection and ranging
LPF	Low-pass filter
OFDR	Optical frequency domain reflectometry
OTDR	Optical time domain reflectometry
PC	Personal computer
PS	Processing system
RBS	Rayleigh backscattered (light)
RF	Radio frequency
RFADC	Radio frequency analog-to-digital converter
RFDC	Radio frequency data converter
RFSoC	Radio frequency system on chip
ROM	Read-only memory
SNR	Signal-to-noise ratio
UART	Universal asynchronous receiver/transmitter
VHF	Very high frequency
VLSI	Very large-scale integration
WT	Wavelet transform
ZIF	Zero intermediate frequency
